# Atmospheric deposition, CO_2_, and change in the land carbon sink

**DOI:** 10.1038/s41598-017-08755-8

**Published:** 2017-08-29

**Authors:** M. Fernández-Martínez, S. Vicca, I. A. Janssens, P. Ciais, M. Obersteiner, M. Bartrons, J. Sardans, A. Verger, J. G. Canadell, F. Chevallier, X. Wang, C. Bernhofer, P. S. Curtis, D. Gianelle, T. Grünwald, B. Heinesch, A. Ibrom, A. Knohl, T. Laurila, B. E. Law, J. M. Limousin, B. Longdoz, D. Loustau, I. Mammarella, G. Matteucci, R. K. Monson, L. Montagnani, E. J. Moors, J. W. Munger, D. Papale, S. L. Piao, J. Peñuelas

**Affiliations:** 1 CSIC, Global Ecology Unit, CREAF-CSIC-UAB, Cerdanyola del Vallès, 08193 Barcelona, Catalonia Spain; 2 CREAF, Cerdanyola del Vallès, 08193 Barcelona, Catalonia Spain; 3 Centre of Excellence PLECO (Plant and Vegetation Ecology), Department of Biology, University of Antwerp, 2610 Wilrijk, Belgium; 4 Laboratoire des Sciences du Climat et de l’Environnement, CEA CNRS UVSQ, 91191 Gif-sur-Yvette, France; 5 International Institute for Applied Systems Analysis, Schlossplatz 1, 2361 Laxenburg, Austria; 6 Global Carbon Project, CSIRO Oceans and Atmosphere, Canberra, ACT 2601 Australia; 7 Sino-French Institute of Earth System Sciences, College of Urban and Environmental Sciences, Peking University, Beijing, 100871 China; 8 Laboratoire de Météorologie Dynamique, Université Pierre et Marie Curie, Paris, 75005 France; 9 TU Dresden, Institut für Hydrologie und Meteorologie, LS Meteorologie, Pienner Str. 23, 01737 Tharandt, Germany; 10 Department of Evolution, Ecology, and Organismal Biology, The Ohio State University, Columbus, Ohio, 43210 USA; 11 Foxlab Joint CNR-FEM Initiative, Via E. Mach 1, 38010 San Michele all’Adige, Italy; 12 Department of Sustainable Agro-Ecosystems and Bioresources, Research and Innovation Center, Fondazione Edmund Mach, 38010 S Michele all’ Adige Trento, Italy; 13 Department of Biosystem Engineering (BioSE), Gembloux Agro-Bio Tech, University of Liege, Liège, 4000 Belgium; 14 Department of Environmental Engineering, Technical University of Denmark (DTU), Lyngby, Denmark; 15 Bioclimatology, Faculty of Forest Sciences and Forest Ecology, University of Göttingen, Büsgenweg 2, 37077 Göttingen, Germany; 16 Finnish Meteorological Institute, Erik Palménin aukio 1, FI-00560 Helsinki, Finland; 17 Department of Forest Ecosystems & Society, Oregon State University, Corvallis, OR 97331 USA; 18 Centre d’Ecologie Fonctionelle et Evolutive CEFE, UMR 5175, CNRS, Université de Montpellier, Université Paul-Valery Montpellier, EPHE, 1919 route de Mende, 34293 Montpellier 5, France; 19 UMR Ecologie et Ecophysiologie Forestières, UMR1137, Inra-Université de Lorraine, Champenoux (F-54280)-Vandoeuvre Les Nancy (F-54500), France; 20 INRA, UMR 1391 ISPA, Centre de Bordeaux Aquitaine, Villenave-d’Ornon, France; 21 Department of Physics, University of Helsinki, P.O. Box 48, FIN-00014 Helsinki, Finland; 22 IBAF - National Research Council of Italy, I-00015 Monterotondo (RM), Italy; 23 ISAFOM - National Research Council of Italy, I-87036 Rende (CS), Italy; 24 School of Natural Resources and the Environment and Laboratory of Tree Ring Research, University of Arizona, Tucson, Arizona USA; 25 Forest Services, Autonomous Province of Bolzano, Via Brennero 6, 39100 Bolzano, Italy; 26 Faculty of Science and Technology, Free University of Bolzano, Piazza Università 5, 39100 Bolzano, Italy; 27 Alterra Wageningen UR, PO Box 47, 6700 AA Wageningen, Netherlands; 28 VU University Amsterdam, Boelelaan 1085, Amsterdam, Netherlands; 29 School of Engineering and Applied Sciences, Harvard University, Cambridge, MA 02138 USA; 30 DIBAF, University of Tuscia, 01100 Viterbo, Italy; 31 Institute of Tibetan Plateau Research, Chinese Academy of Sciences, Beijing, 100085 China

## Abstract

Concentrations of atmospheric carbon dioxide (CO_2_) have continued to increase whereas atmospheric deposition of sulphur and nitrogen has declined in Europe and the USA during recent decades. Using time series of flux observations from 23 forests distributed throughout Europe and the USA, and generalised mixed models, we found that forest-level net ecosystem production and gross primary production have increased by 1% annually from 1995 to 2011. Statistical models indicated that increasing atmospheric CO_2_ was the most important factor driving the increasing strength of carbon sinks in these forests. We also found that the reduction of sulphur deposition in Europe and the USA lead to higher recovery in ecosystem respiration than in gross primary production, thus limiting the increase of carbon sequestration. By contrast, trends in climate and nitrogen deposition did not significantly contribute to changing carbon fluxes during the studied period. Our findings support the hypothesis of a general CO_2_-fertilization effect on vegetation growth and suggest that, so far unknown, sulphur deposition plays a significant role in the carbon balance of forests in industrialized regions. Our results show the need to include the effects of changing atmospheric composition, beyond CO_2_, to assess future dynamics of carbon-climate feedbacks not currently considered in earth system/climate modelling.

## Introduction

Terrestrial ecosystems are key components of the global carbon cycle. Since the 1960s, they have been sequestering an average of about 30% of the annual anthropogenic CO_2_ emitted into the atmosphere^1^. The increase in atmospheric CO_2_ concentration (hereafter CO_2_) affects the terrestrial biosphere in multiple ways: warming the climate (radiative effect)^2^, increasing photosynthesis (CO_2_ fertilization), decreasing transpiration by stimulating stomatal closure (leading to increased water-use efficiency) and changing the stoichiometry of carbon, nitrogen and phosphorus (C:N:P) in ecosystem carbon pools^3^. Although Earth system models simulate rising CO_2_ to make a significant contribution to increasing plant productivity and C storage, empirical evidence remains elusive^4^. This uncertainty is evidenced by the fact that many studies reporting observations of large-scale increases in productivity (or “greening”) in the Northern Hemisphere have attributed these increases to different contributing mechanisms. These mechanisms include the CO_2_-fertilization effect (i.e., more CO_2_ leads to more photosynthesis), the lengthening of the growing season due to higher winter, spring or autumn temperatures^5^, nitrogen deposition^6^, recovery from acidic deposition^7^ and afforestation or forest regrowth^8^. Here, we combine all these potential drivers to reveal the dominant drivers of the increase in C fluxes across 23 northern hemisphere forests.

Many experimental studies have shown that productivity increases when ecosystems are exposed to artificially elevated CO_2_^9^. However, despite being highly valuable, these experiments do not resemble natural conditions because they cannot capture the long-term responses of mature forest ecosystems to gradually increasing CO_2_ concentrations. In this sense, a CO_2_-fertilization effect has not yet been firmly established in terrestrial ecosystems so far. Positive effects of increasing CO_2_ on productivity are, in fact, only expected when other factors are not limiting growth (e.g., water and nutrient availability)^10^. Some studies attributed increased ecosystem water-use efficiency to the reduction in transpiration resulting from increased CO_2_^11^, but they have not always been able to link it to enhanced plant growth^10, 12^.

Detecting a fertilization effect from increasing CO_2_ in terrestrial ecosystems is difficult because many other factors, that also alter ecosystem productivity trends, are changing concurrently. One of such confounding variable is the physical change in climate, which alters ecosystem productivity directly by impacting the ecosystem C cycle, and indirectly by increasing nutrient mineralization rates and the length of the growing season. Atmospheric deposition of nitrogenous and sulphurous compounds (N and S deposition) also alter ecosystem processes.

There is strong evidence indicating that N deposition has increased the terrestrial C sink^13–16^. By acidifying the soil, sulphur deposition can reduce plant growth and increase leaching of soil nutrients needed by plants^17^. Some studies have shown that reduced S deposition is associated with recovery in tree growth through increased net photosynthesis and stomatal conductance^18^, however, the role of S deposition on forest productivity and C storage has rarely been explored^19^. In Europe and North America, air-quality policies to reduce emissions of pollutants (SO_2_ and NOx) have proven effective and have decreased acidic deposition (mainly SO_4_^2−^ and NO_3_^−^) substantially since 1980^20, 21^. The reduction in acidic deposition of both N and S should lead to a slow recovery of forests to a pre-acid deposition state. On the other hand, decreasing N deposition could also slow down forest growth and C sequestration once previously accumulated soil N is used up and N again becomes a limiting nutrient^15, 22^.

Here, we test the hypothesis that gross primary production (GPP), ecosystem respiration (Re) and the net C-sink strength (net land-atmosphere CO_2_ flux) or net ecosystem production (hereafter NEP), have accelerated during the last two decades because of the increased atmospheric CO_2_ concentrations and temperature, and because of the recovery from high loads of S deposition in Europe and North America. However, decreasing atmospheric deposition of N may have constrained productivity. We expected these deposition reductions to have modulated the biogeochemical effects of rising CO_2_.

To test our hypothesis, we used long-term eddy-covariance observations of NEP, derived GPP, and Re from 23 temperate and boreal forest sites distributed across Europe and the USA (see Supplementary Fig. S1). For these 23 forests, we also used remotely sensed maximum leaf area index (LAI) as a proxy for canopy development, derived from the AVHRR GIMMS NDVI3g data set^23^. Data for predictor variables were acquired from: i) gridded maps for wet N and S deposition for Europe (European Monitoring and Evaluation Programme)^24^ and the USA (National Atmospheric Deposition Program)^25^, and ii) historical climate data from the Climatic Research Unit (TS v.3.21) for time series of temperature, precipitation^26^ and the Standardized Precipitation-Evapotranspiration Index (SPEI) — a measure of meteorological drought^27^.

## Results

### Individual trends of NEP, GPP, Re, LAI and predictor variables

**Figure 1:**
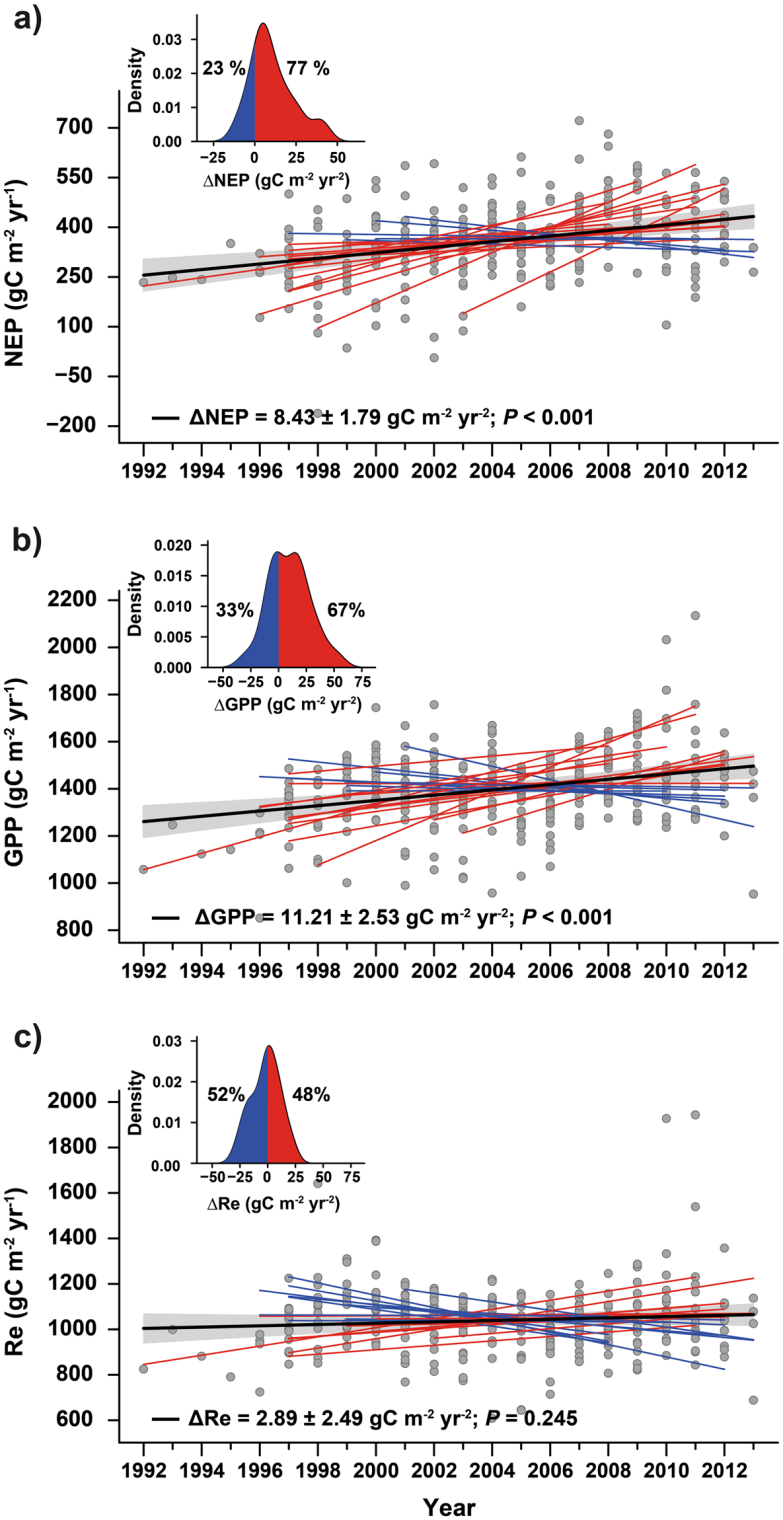
Long-term trends in C fluxes for 23 forests (1992–2013). Most of the forests presented increasing trends in (**a**) NEP and (**b**) GPP, whereas (**c**) respiration remained fairly constant. The percentage of forests with increasing NEP was statistically higher (*P* = 0.001) than the percentage of forests with decreasing NEP, but the percentage of forests where GPP tended to increase was not statistically different (*P* = 0.28) from those with decreasing GPP. Red and blue lines indicate forests with increasing and decreasing trends, respectively, and black lines indicate the average trends. The shaded area indicates the standard error of the average trend. Grey dots indicate forest-year observations, and all values were adjusted to the same mean to remove forest-specific variability. The inset shows the modelled distribution of the trends using kernel-density estimation, indicating the percentage of forests with increasing and decreasing trends. See Methods for further information on the methodology used to calculate the trends. All data came from eddy-covariance towers.

**Figure 2:**
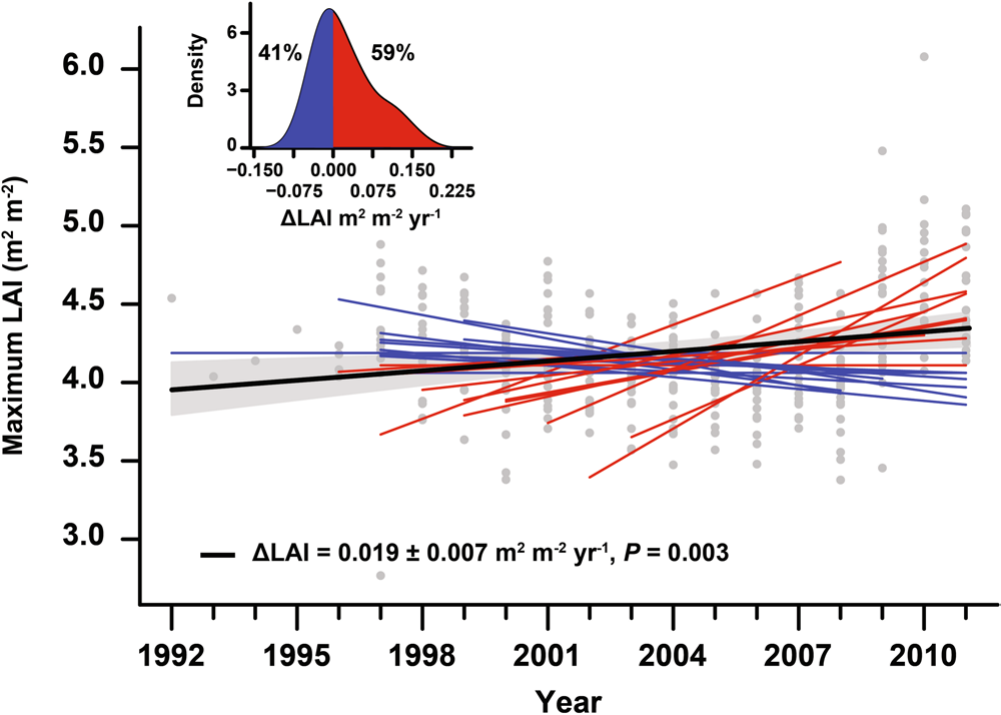
Trends in forest maximum LAI. Red and blue lines indicate forests with increasing and decreasing trends, respectively, and the thick black line indicates the average trend. The shaded area indicates the standard error of the average trend. Grey dots indicate forest-year observations, and all values were adjusted to the same mean to remove forest-specific variability. The inset shows the modelled distribution of the trends using kernel-density estimation.

Averaged across the 23 temperate and boreal forests, annual NEP and GPP increased (mean ± SE) by 8.4 ± 1.8 and 11.2 ± 2.5 g C m^−2^ yr^−1^, respectively, during the studied period (*P* < 0.001). The increase corresponds to an annual increase of 1.1% in both C fluxes, consistent in magnitude with growth rates reported in previous studies^28^ and simulated by global models in response to rising CO_2_ only^29^. NEP increased over time in 18 of the 23 forests; for 11 of these 18 the increase was statistically significant at *P* < 0.05 (Fig. 1a inset, Table 1). Bootstrapping analysis show that forests with increasing NEP clearly outnumbered those in which NEP did not increase (*P* = 0.001; Fig. 1a). Similarly, GPP increased over time in 14 of the 23 forests, with eight forests presenting statistically significant trends at *P* < 0.05 (Fig. 1b, Table 1). Re of individual forests increased by 2.9 ± 2.5 g C m^−2^ yr^−2^, but this signal was not statistically significant (*P* = 0.25). This led NEP to increase slightly less than GPP, (Fig. 1c and Table 1). Additionally, we found maximum LAI derived from satellite data to exhibit an overall increasing trend (0.019 ± 0.007 m^2^ m^−2^ yr^−1^, *P* = 0.003) across the 23 forests (Fig. 2). Maximum LAI increased for 14 of the 23 forests, being a statistically significant increase for 5 of these 14 forests.

**Table 1:** Summary of the main characteristics of the forests and the trends presented by NEP, GPP, Re, and maximum LAI.

Forest	Code	Climate	Forest Type	Age	Maturity Age	Corrected Mat. Age	Initial year	Finalyear	Y	NEP TS Trend	*P*	GPP TS Trend	*P*	Re TS Trend	*P*	LAITS Trend	*P*
Brasschaat^1^	BE-Bra	Temp	M	80	90	0.89	1997	2011	14	17.5 ± 7.5	0.0773	21.4 ± 13.9	0.0313	9.7 ± 19.8	0.2556	0.000 ± 0.008	0.6329
Castelporziano^2^	IT-Cpz	Temp	EB	61	75	0.81	1997	2008	10	2.8 ± 6.9	0.3603	−12.8 ± 16.9	0.7629	−22.1 ± 12.8	0.8145	0.100 ± 0.010	0.0173
Collelongo^3^	IT-Col	Temp	DB	118	95	1.24	1997	2012	12	4.1 ± 9.6	0.2686	16.8 ± 11.9	0.1219	8.5 ± 6.4	0.0574	−0.014 ± 0.015	0.8299
Hainich^4^	DE-Hai	Temp	DB	275	95	2.89	2000	2012	13	−7.3 ± 4.6	0.9197	−11.2 ± 7.3	0.8502	−6.3 ± 5.9	0.7489	0.047 ± 0.018	0.0466
Harvard^5^	US-Ha1	Temp	DB	81	75	1.07	1992	2011	20	12.6 ± 5.9	0.0372	34.7 ± 5.1	<0.0001	20.2 ± 9.6	0.0075	0.000 ± 0.005	0.6539
Hesse^6^	FR-Hes	Temp	DB	43	95	0.45	1996	2010	15	26.4 ± 11.0	0.0374	18.3 ± 15.4	0.1381	0.5 ± 15.3	0.5000	0.017 ± 0.007	0.2737
Howland MT^7^	US-Ho1	Temp	EC	109	90	1.21	1996	2008	13	6.5 ± 2.8	0.0293	−5.8 ± 7.5	0.7489	−16.2 ± 7.2	0.9364	−0.050 ± 0.008	0.9934
Howland F^7^	US-Ho2	Temp	EC	109	90	1.21	1999	2009	11	5.4 ± 4.8	0.1751	7.7 ± 8.2	0.2667	2.6 ± 11.0	0.3202	−0.025 ± 0.011	0.7621
Hyytiala^8^	FI-Hyy	Bor	EC	47	90	0.52	1997	2012	16	6.2 ± 2.5	0.0172	14.7 ± 4.0	0.0017	10.4 ± 3.5	0.0051	0.000 ± 0.004	0.5201
Lavarone^9^	IT-Lav	Temp	EC	120	90	1.33	2003	2012	10	41.8 ± 10.3	0.0100	37.2 ± 12.8	0.0159	−2.7 ± 5.1	0.7042	0.114 ± 0.022	0.1008
Le Bray^10^	FR-LBr	Temp	EC	38	90	0.42	1997	2008	11	7.2 ± 18.8	0.4381	10.8 ± 25.6	0.3777	−18.3 ± 12.6	0.8935	−0.033 ± 0.014	0.8465
Loobos^11^	NL-Loo	Temp	EC	88	90	0.98	1997	2012	16	21.5 ± 4.9	0.0009	−6.0 ± 4.2	0.9186	−27.1 ± 5.9	0.9991	−0.017 ± 0.008	0.6559
Metolius^12^	US-Me2	Temp	EC	64	90	0.71	2002	2012	11	13.4 ± 9.8	0.1379	29.0 ± 13.7	0.0806	10.6 ± 13.4	0.2667	0.156 ± 0.028	0.0866
Morgan Monroe^7^	US-MMS	Temp	DB	70	75	0.93	1999	2013	15	−2.6 ± 3.9	0.8619	−1.8 ± 5.5	0.6897	0.7 ± 5.0	0.5000	−0.041 ± 0.009	0.8677
Niwot ridge^13^	US-NR1	Bor	EC	98	90	1.09	1999	2010	12	1.9 ± 2.8	0.4185	−0.1 ± 3.4	0.5000	−1.3 ± 2.3	0.6341	0.060 ± 0.005	0.0166
Park Falls^14^	US-PFa	Temp	DB	44	65	0.68	1997	2013	16	9.6 ± 3.7	0.0172	0.1 ± 4.3	0.4820	−12.1 ± 6.2	0.9425	−0.008 ± 0.008	0.5873
Puechabon^2^	FR-Pue	Temp	EB	66	75	0.88	2001	2013	13	−10.3 ± 6.6	0.9197	−28.4 ± 13.3	0.9502	−18.5 ± 8.7	0.9880	0.114 ± 0.014	0.0108
Renon^15^	IT-Ren	Bor	EC	90	75	1.20	1998	2011	13	37.9 ± 5.3	0.0001	51.9 ± 8.7	0.0006	10.2 ± 6.3	0.0636	0.030 ± 0.007	0.1202
Sodankyla^16^	FI-Sod	Bor	EC	75	90	0.83	2000	2012	13	−0.2 ± 1.6	0.5000	−2.8 ± 7.1	0.5243	0.6 ± 6.8	0.4757	0.047 ± 0.005	0.1346
Soroe^17^	DK-Sor	Temp	DB	78	95	0.82	1997	2009	13	27.3 ± 4.8	0.0004	22.9 ± 8.4	0.0164	−0.2 ± 8.8	0.5000	−0.017 ± 0.007	0.7336
Tharandt^18^	DE-Tha	Temp	EC	117	90	1.30	1997	2013	17	−1.2 ± 3.6	0.6446	16.1 ± 8.3	0.0383	20.4 ± 6.7	0.0178	−0.025 ± 0.014	0.6730
UMBS^19^	US-UMB	Temp	DB	79	65	1.22	1999	2012	14	5.4 ± 3.1	0.0080	−3.5 ± 5.4	0.7444	−10.4 ± 4.5	0.9373	0.058 ± 0.005	0.0017
Vielsalm^20^	BE-Vie	Temp	M	83	95	0.87	1996	2008	13	17.0 ± 6.9	0.0062	15.1 ± 6.8	0.0120	−0.3 ± 7.2	0.5243	0.2330	

Trends were computed using the robust Theil-Sen slope estimator. *P* indicates a one-tailed *P* (H1: trend > 0). Corrected maturity age was calculated by dividing the mean stand age by the logging maturity tree age as described by Stokland *et al*.^60^ for average productivity classes. Abbreviations: Y, years; TS, Theil-Sen; Clim for Climate; Temp, temperate; Bor, boreal; for, Forest type; M, mixed; E, evergreen; D, deciduous; B, broadleaved; C, coniferous; EC, eddy covariance. Upperscript numbers indicate reference numbers, see additional References in Supplementary Material.

**Figure 3:**
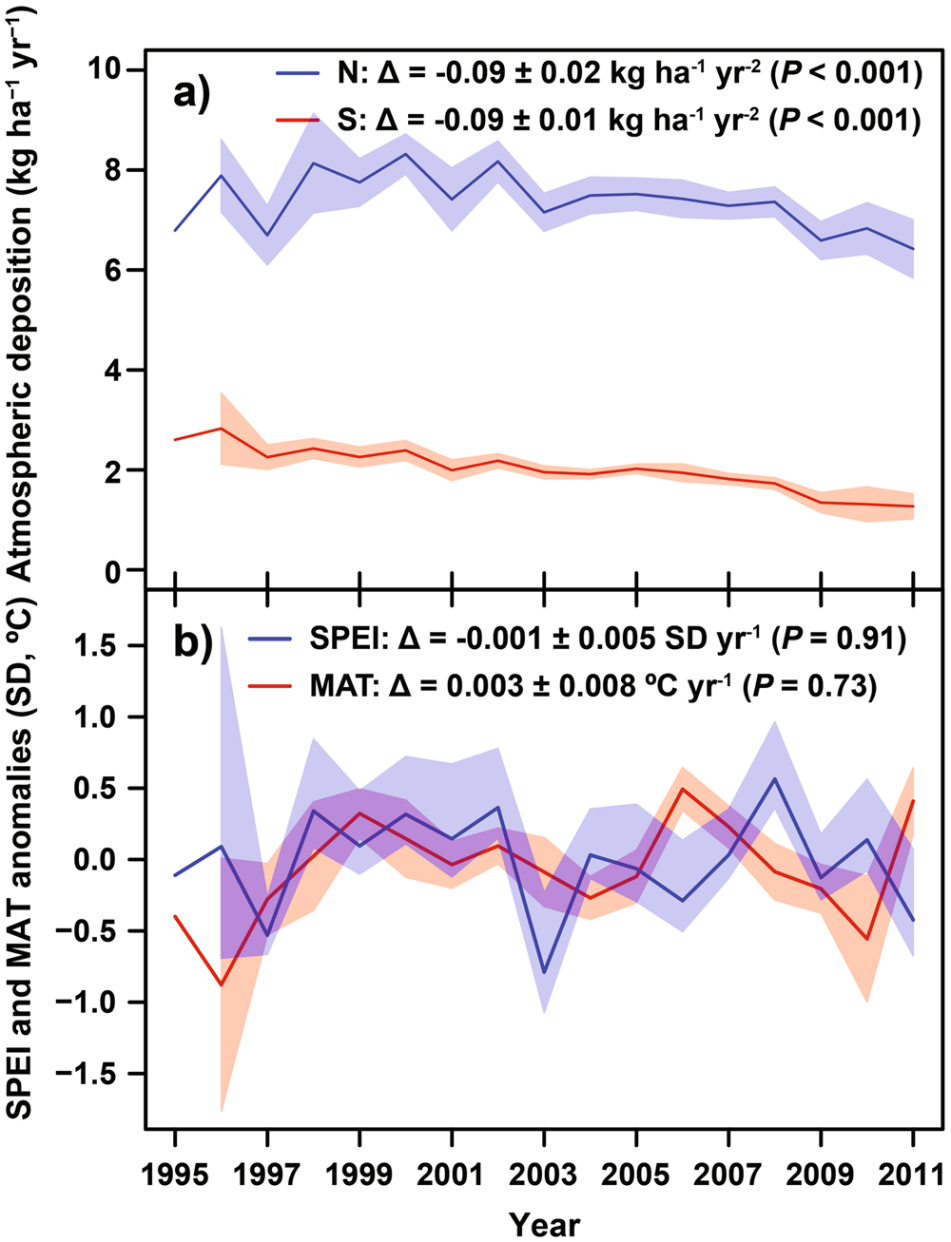
Temporal evolution and trends in N and S deposition, mean annual temperature (MAT), SPEI for the 23 forest sites (1995–2011). Trends were calculated using GLMMs with random slopes, with the forest as a random effect and year as a fixed effect. Models also used an ARMA (1,0) autocorrelation structure. Shading indicates the 95% confidence intervals of the means (calculated as 1.96 times the standard error of the mean). See Methods for further details.

Across the 23 forests in Europe and USA, CO_2_ increased on average by 2.0 ± 0.1 ppm yr^−1^, but neither mean annual temperature (MAT) nor the hydric conditions (SPEI) changed significantly over the same period (Tables S1 and S2, Fig. 3). This apparent climatic stability may partly result from the relatively short time series analysed (10 to 20 years). Conversely, N and especially S deposition exhibited strong and in generally monotonic downward trends from 1995 to 2011 across the 23 forests. On average, N deposition decreased by 1.1% annually (−0.09 ± 0.02 kg N ha^−1^ yr^−2^; *P* < 0.001) and S deposition by 4.6% annually (−0.09 ± 0.01 kg S ha^−1^ yr^−2^; *P* < 0.001) (Tables S1 and S2, Fig. 3).

### Spatial variability in individual trends of NEP, GPP, Re and LAI

**Figure 4:**
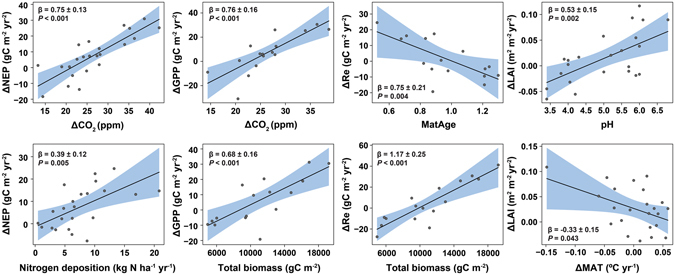
Partial residual plots showing significant relationships found between predictors and C-flux trends in the 23 forests (ΔNEP/Δt, ΔGPP/Δt and ΔRe/Δt) and ΔLAI/Δt. Model summaries can be found in Supplementary Information section 1. Corrected maturity age (MatAge) was calculated by dividing the mean stand age by the logging maturity tree age as described by Stokland *et al*.^60^ for average productivity classes. See Methods for more information on the calculation of the corrected logging maturity age.

A regression analysis of the individual trends (see Supplementary Information 1 and Fig. 4) indicates that the annual trends of NEP and GPP were mostly positively correlated with the increasing trend of CO_2_ (Fig. 4). Forests with larger standing biomass presented more positive trends in GPP and especially Re but not in NEP. Instead, trends of NEP were higher in forests with higher N deposition. Within forests, Re increased with positive trends of MAT, which, consequently, reduced trends of NEP. Older forests presented lower or more negative trends of Re than young forests (Supplementary Information 1, Fig. 4). Our analysis did not show significant statistical associations between C flux trends in individual forests and other possible factors (e.g., trends in S deposition, see Supplementary Fig. S2) or forest characteristics, such as mean annual precipitation, soil pH, or leaf type and habit. However, trends in maximum LAI presented a positive association with soil pH and a negative association with trends in MAT (Supplementary Information 1 and Fig. 4).

### Drivers of trends in C fluxes: temporal contributions and sensitivities

**Figure 5:**
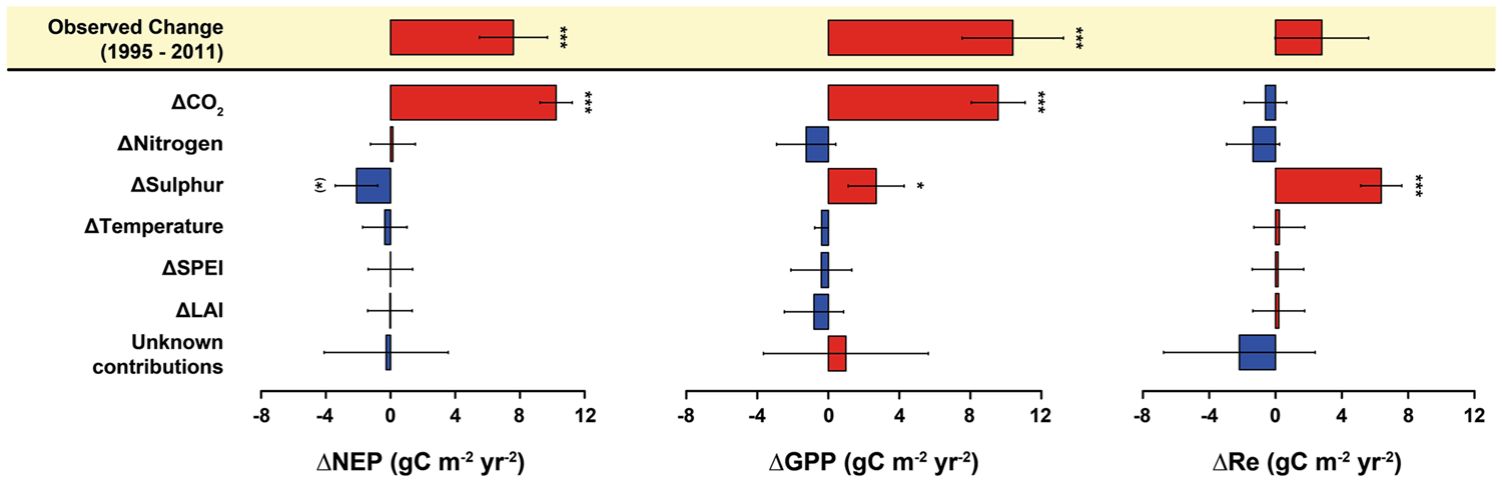
Temporal contribution of the predictor variables on NEP, GPP and Re, for the period 1995–2011. Models (see Supplementary Information, section 2.1.1–2.1.3) suggest that increasing CO_2_ is the main contributor to the observed increases in NEP and GPP. The difference between the modelled contributions and the observed trends (yellow shaded) has been considered as an unknown contribution to the temporal variation in C fluxes. The temporal variations of the predictors are shown in Fig. 3. Error bars indicate standard errors. Units are ppm for CO_2_, kg ha^−1^ yr^−1^ for S and N deposition, °C for temperature and standard deviation for SPEI. Data for forest C fluxes came from eddy-covariance towers. Error bars indicate standard errors. See Methods for information about the methodology used to calculate the contributions. Significance levels: ^(^*^)^ *P* < 0.1; *P < 0.05; **P < 0.01; ***P < 0.001.

We used generalized linear mixed models (GLMMs) and model averaging to attribute the temporal trends of NEP, GPP and Re to changes in CO_2_, N and S deposition rates, MAT, SPEI, LAI and their interactions by calculating the difference between trends predicted by the full model and those maintaining one of the temporal covariates (i.e., anomalies) constant at a time (see Methods for further details). We found that increasing CO_2_ is the only predictor systematically associated with the observed increase in both NEP and GPP over time (Fig. 5). For each ppm increase in atmospheric CO_2_ concentration, NEP and GPP increased by 4.81 ± 0.52 and 4.49 ± 0.75 g C m^−2^ yr^−1^, respectively (Table 2). Conversely, increasing CO_2_ had no statistically significant association with increasing Re (Fig. 5) despite the normally close relationship between Re and GPP^15^. The statistical models also show that the decrease of S deposition during the period of flux measurements at both European and USA forests (Fig. 3 and Table S2) has also affected the CO_2_ fluxes in these forests (Fig. 5).

**Table 2:** NEP, GPP and Re mean sensitivity to predictors for the 23 forests for the period 1995–2011.

	NEP	*P*	GPP	*P*	R_e_	*P*
CO_2_ (ppm)	**4.81 ± 0.52**	<0.0001	**4.49 ± 0.75**	<0.0001	−0.29 ± 0.60	0.3183
Nitrogen (kg ha^−1^ yr^−1^)	−1.64 ± 15.96	0.4593	14.41 ± 19.39	0.2029	15.62 ± 18.65	0.2044
Sulphur (kg ha^−1^ yr^−1^)	**24.45 ± 15.42**	0.0616	**−31.24 ± 18.52**	0.0511	**−74.01 ± 16.02**	<0.0001
Temperature (K)	−126.74 ± 408.16	0.4182	−137.36 ± 415.44	0.3716	80.46 ± 592.16	0.4464
SPEI (SD)	13.67 ± 2225.46	0.4976	645.30 ± 6256.23	0.4593	−238.29 ± 3255.54	0.4711
LAI (m^2^ m^−2^)	−1.80 ± 66.73	0.4893	9.37 ± 76.01	0.4514	28.86 ± 104.40	0.3930

Sensitivities (units of change in the response variable for each unit of change in the predictor) were calculated by dividing the temporal contributions of the predictor (Fig. 5) by the trend of the predictors (Figs 2 and 3, Table S2). Nitrogen and sulphur refers to atmospheric deposition, and temperature to mean annual air temperature. Errors were calculated by error propagation^63^. NEP, GPP and Re units are g C m^−2^ yr^−1^. Bold type indicates statistically significant sensitivities.

The reduction in S deposition was associated with a net decrease in NEP (NEP sensitivity: 24.45 ± 15.42 g C m^−2^ yr^−1^ for each kg S ha yr^−1^), likely because of a larger (*P* = 0.038) increase of Re than GPP as forests recover from past S deposition. The sensitivity of Re and GPP to each kg S ha^−1^ yr^−1^ is −74.01 ± 16.02 and −31.24 ± 18.52 g C m^−2^ yr^−1^, respectively. These results imply that the reduction in S deposition reduced the positive effect of CO_2_ fertilization on NEP by 21 ± 13%. However, the reduction in N deposition tended to reduce both GPP and Re, but this effect of reduced N deposition was not statistically significant (Fig. 5). The sensitivity of Re and GPP to each kg N ha^−1^ yr^−1^ is 15.62 ± 18.65 and 14.41 ± 19.39 g C m^−2^ yr^−1^, respectively (see Fig. 5 and Table 2). Using past N and S deposition, i.e. the cumulative totals of the previous 5 years, did not improve our models according to the variance explained, the second-order Akaike Information Criterion (AICc) and the Bayesian Information Criterion (BIC).

**Figure 6:**
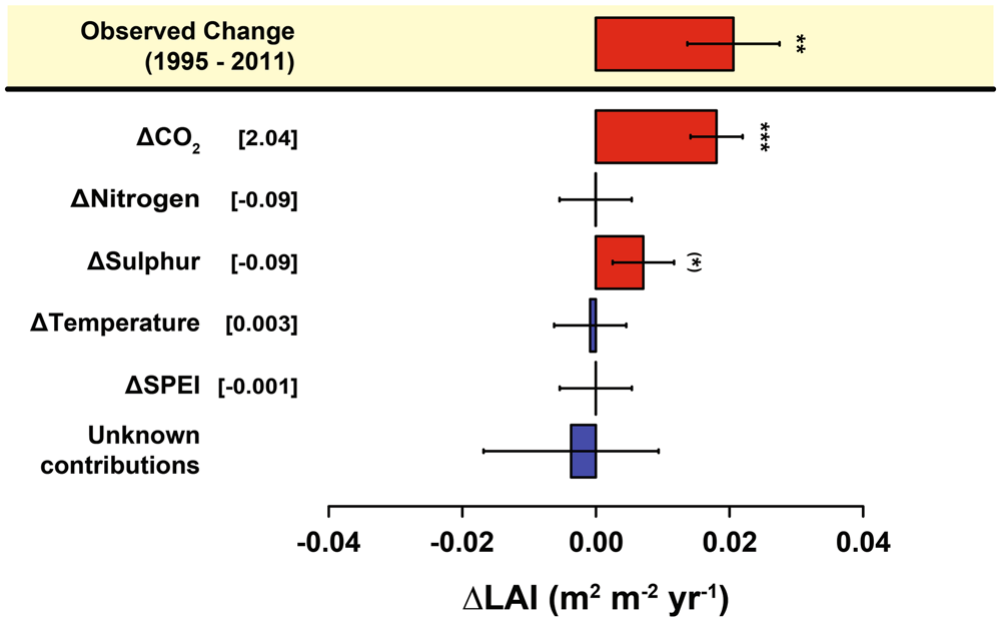
Temporal contribution of the predictor variables. The model (Supplementary Information, section 2.1.4) suggested that increasing CO_2_ is the main contributor to the observed increases in LAI. The difference between the modelled contributions and the observed trends has been considered as an unknown contribution to the temporal variation LAI. The temporal variations of the predictors are shown in square brackets. Error bars indicate standard errors. Units are ppm for CO_2_, kg ha^−1^ yr^−1^ for S and N deposition, °C for temperature and standard deviations for SPEI. Error bars indicate standard errors. See Methods for information about the methodology used to calculate the contributions. Significance levels: ^(^*^)^ *P* < 0.1; *P < 0.05; **P < 0.01; ***P < 0.001.

The combined effect of reductions in S and N suggest that the positive effect gained from reduced S deposition on GPP and Re was offset by 47 ± 68% and 21 ± 25%, respectively, due to the opposite effect of reduced N deposition. Trends in climate (MAT and SPEI) did not influence trends in CO_2_ fluxes over the timeframe of this study (1995–2011). Using temperature and SPEI from the warm half of the year (April – September) in our models did not show any greater influence of climate on C flux trends either (see Models 2.2.1–2.2.3 in Supplementary Information). In addition, the increasing LAI was not correlated with the changing C fluxes (Fig. 5, Table 2). Finally, the model used to detect potential causes for the increased LAI showed, again, that rising CO_2_ and decreased S deposition were the only factors of significant importance for temporal changes across the 23 forests (Fig. 6).

## Discussion

### Empirical evidence of CO_2_ fertilization effect

Even though our statistical analyses do not directly prove causality, the results provide consistent empirical evidence that support the dominant role of the CO_2_-fertilization effect in explaining the current positive NEP trends at local and possibly regional scales (Fig. 5). The results support our hypotheses, which were based on the state of the art from earlier studies, refined with respect to other drivers (S deposition), and corroborated by careful attribution of variances with GLMM. The results indicate a relatively strong CO_2_ fertilization effect given the somewhat short span of CO_2_ increases in our data set (increasing by 13–47 ppm during the study period, depending on the forest and database, see Table 1 and Supplementary Table S2). This increase is relatively small compared to the increases applied in free-air CO_2_-enrichment experiments (typically 475–600 ppm^9^, i.e., a step change in CO_2_ of ~100–200 ppm).

Our results also show a much higher sensitivity of NEP to CO_2_ of 4.81 ± 0.52 g C m^−2^ y^−1^ ppm^−1^ when compared to the sensitivity obtained from CO_2_-enrichment FACE experiments of 1 g C m^−2^ y^−1^ ppm^−1^, which is a 10% increase in net primary production (assuming an average of 1000 g C m^−2^ y^−1^) for a step increase of 100 ppm^30, 31^. This discrepancy may be related to the differences between small gradual increases in CO_2_ seen in the environment versus large stepwise increases in CO_2_ manipulative experiments. It has been suggested that the progressive nutrient limitation makes CO_2_ fertilization be stronger at lower CO_2_ increases and become saturated at higher levels like the ones in experiments (e.g. 600 ppm)^32, 33^.

Increasing CO_2_ can enhance photosynthesis by increasing the rate of carboxylation and reducing losses from photorespiration^34^. Increasing CO_2_ might also decrease stomatal conductance, leading to increased water-use efficiency^11, 35^, but the relationship between increasing water-use efficiency and higher plant growth and net C uptake in ecosystems is still controversial^10, 11^. Because tissues with high C:N ratio are more difficult to decompose than tissues with lower C:N ratio, the increase in litter C:N ratio due to increased CO_2_^36^ might reduce heterotrophic respiration^37, 38^ and, therefore, increase the C-sink strength, at least during a transient period. Elevated CO_2_, though, could also stimulate root exudation, thereby increasing the priming effect and reduce soil C stocks^39^. However, those counteracting mechanisms seem to offset each other in our forests, resulting in no significant change in Re. Nonetheless, despite possible counteracting mechanisms, elevated CO_2_ seems to be responsible for the increases in terrestrial photosynthesis and C sequestration during the last decades.

### Recovery from high loads of acid deposition in Europe and USA

The negative effect of the reduction in S deposition on NEP is the consequence of differences in recovery of the gross fluxes from previously higher rates of acid deposition, i.e. the recovery of Re is stronger than that of GPP (Fig. 5 and Table 2). We postulate that this follows from a chain of processes during recovery from soil acidification. A reduction in S deposition, in our case combined with a reduction in N deposition, typically increases soil pH, which, in turn, increases microbial activity^40^, thereby increasing heterotrophic respiration and thus nutrient mineralization and availability^41^, with implications for both GPP and Re. The potential increase in pH and nutrient availability, during a recovery phase after high S deposition, can enhance photosynthesis and tree growth^18^ in a second step, i.e. when nutrient availability has considerably increased. Even if pH remains unaltered, reduced acid input reduces aluminium release in soil and, therefore, less damage to roots occur, potentially increasing productivity^42^. While higher microbial activity in response to reduced S deposition increases respiration, the associated higher nutrient availability can in turn reduce C allocation to root symbionts^43^ and to free living heterotrophs via exudates, therefore ultimately reducing heterotrophic respiration^44^. These two opposing mechanisms may compensate each other to some degree after some time. The stronger positive response of Re to declining S deposition than of GPP (Fig. 5) suggests a stronger contribution of the increase in microbial respiration, following recovery, than a possible reduction of respiration due to decreased belowground C allocation. Nonetheless, the results obtained here are quite surprising given the relatively small change observed in S deposition that, according to soil models, would have a low impact on soil pH and aluminium release^45^.

In addition to soil biochemical impacts, reduction in sulphurous pollutants affect optical properties of the atmosphere by reduced secondary aerosol (SOA) formation. S emissions lead to higher aerosol densities, which affect photosynthesis in two opposite ways: by reducing total light inputs, photosynthesis would be reduced, but by increasing the ratio of diffuse over direct radiation, photosynthesis in deeper layers of the canopy would increase^46, 47^. We speculate, from the overall negative effect of S deposition on GPP, that the disadvantage from decreasing diffuse light fraction because of reduced S emissions is lower than the positive effect due to the recovery from acidification of an ecosystem.

The impacts of N deposition on forest ecosystem C cycling have been widely studied. Reduced heterotrophic respiration is a general response to N deposition, possibly through an enhanced stabilization of soil organic matter, altered plant carbon allocation patterns and shifts in the saprotrophic community^48^. Nitrogen fertilization increases aboveground production in young forests, while decreasing autotrophic and heterotrophic respiration^49^, and hence potentially enhancing ecosystem C uptake (or increase C stocks)^15, 22^. However, in N limited ecosystems and young stands, low levels of N deposition can increase respiration because of enhanced biomass production and the associated increase in maintenance and growth respiration^14, 44^. N deposition - where N is a limiting nutrient - will increase net primary production^50^ through its effect on photosynthesis^51^ and possibly by the above-mentioned increasing C allocation to wood production at the expense of symbionts and exudates^43^. Our analysis of spatial variability supported these hypotheses (Supplementary Information, models in section 1 and 2), but our analysis of temporal variability indicated that decreasing N deposition had no statistically significant effect on the trend in NEP, because the small effect of reducing both Re and GPP at the same time (Fig. 5). N deposition rates have been relatively high during several years and the recent decrease (in percentage around a quarter of the decrease in S deposition, see Fig. 3) may have not been large enough to significantly alter C fluxes in forest ecosystems. On the other hand, N is efficiently accumulated and kept in the ecosystem’s internal cycle^52^, thereby protecting it from leaching, whereas this is not usually the case for sulphate in acidic soils^53^.

### Small effects of decadal-scale climate change on the carbon balance

In the 23 forests studied, temperatures and drought did not significantly change and, therefore, could not be responsible for the observed trends in C fluxes. The estimated effect of temperature and drought on CO_2_ fluxes was clearly small compared to the effects of increasing CO_2_ and decreasing S deposition. These results suggest that, during the studied period, availability of CO_2_ and nutrients and stoichiometric changes have exerted a stronger impact on the terrestrial C balance than the changing climate^6^. Nonetheless, given the small increase in temperatures and droughts during the study period, we cannot rule out the possibility that climate change might have larger effects on C fluxes in the future. Larger datasets, including longer time series comprising other geographical regions (i.e., Asia, South America, Africa…) and covering the main biomes of the world, are necessary to correctly answer this question and to better assess the effect of atmospheric deposition on terrestrial C balance.

### Changing land carbon sinks

Multiple drivers are affecting the C budget of terrestrial ecosystems in several ways. Increasing atmospheric CO_2_ concentrations have increased the land C sink by enhancing GPP more than ecosystem respiration. The reduction in S deposition rates is severely altering the C balance by enhancing photosynthesis and ecosystem respiration but in a decoupled manner. In addition, the reduction of N deposition rates in developing countries may soon present a significant effect on forest C balances by reducing both photosynthesis and respiration. However, trends in N and S deposition are divergent depending on the region of the world considered: while S deposition is mainly decreasing in western countries, fossil fuel burning is increasing S deposition rates in Asia^54^. On the other hand, N deposition is expected to approximately double current levels by 2050 globally^55^. Hence, the trends observed for the forests studied here may take place at different regions at different times in the following decades, unless other nutrient imbalances (e.g., limitation of phosphorus) completely change the response of ecosystems^3^.

It is far from certain whether terrestrial ecosystems will continue to respond positively to increasing CO_2_, will saturate, or will eventually reach a tipping point beyond which respiration and the release of greenhouse gases exceed production. Stoichiometric imbalances and the limitation of key nutrients such as nitrogen and phosphorus^3, 6, 56^ may already be acting as limiting factors for enhanced C sequestration. Given these observed complex relationships, partly compensating effects of multiple drivers on the gross C fluxes, GPP and Re, with apparently differing dynamic behaviour, accurate prediction of the future net C sink is complex. It will require biospheric models that include realistic parameterisations of the various biochemical responses of C sequestration processes obtained from real field conditions and experiments. Further, this study shows the need to go beyond climate and CO_2_ to characterize the strength of the land sink, and the future evolution of the carbon-climate feedback. Biospheric and earth system models will need to develop processes to address the effects of additional atmospheric pollutants.

## Materials and Methods

### Data sets

#### Carbon fluxes

We downloaded Level-4 CO_2_ flux data collected by eddy-covariance towers from the Euroflux (GHG-Europe) and Ameriflux databases. When Level-4 data were not available, we downloaded gap-filled Level-2 data and checked for the homogeneity of the time series. In all cases, time series were either Level-2 or Level-4. Level 4 data are obtained after applying u* filtering, gap-filling and partitioning following Reichstein *et al*., (2005). Level-2 data are provided by the PIs, half-hourly, not gap-filled or filtered but quality checked by the PIs. This data was then processed using the Eddy covariance gap-filling & flux-partitioning tool from the Max Planck Institute webpage (http://www.bgc-jena.mpg.de/~MDIwork/eddyproc/) to be equivalent to Level-4 data, also following Reichstein *et al*., (2005). The data used in this study have been harmonized in terms of processing but the fluxes calculation is still heterogeneous because it is performed by the responsible staff of every forest. We also used a global forest database updated in 2013 with data up to 2010^6^ to obtain ancillary data (e.g., stand age and standing biomass) and for comparing CO_2_ flux measurements. We selected 23 forests for which at least 10 years of CO_2_ flux measurements were available. All forests were in the Northern Hemisphere between 39 and 68 °N (see Supplementary Fig. S1), and the years of measurement ranged from 1992 to 2013. These forest sites were selected because they are the longest running flux sites with 10 or more years of data available between 1992 and 2013. The selected forests had no indication of major disturbances or strong management practices which are known to alter C fluxes (in contrast to the typical situation for grasslands or croplands). We also extracted information for all forests about leaf type and habit (evergreen/deciduous), and the age of the stand at the time of the measurements. Soil pH was extracted from the ancillary data of the forests when possible (17 forests), but when not available, pH was assessed using data from the Harmonized World Soil Database^57^ (4 forests) and published literature reviews^6^ (2 forests).

#### Remotely sensed LAI data

We calculated the maximum annual LAI for the 23 forests from the GIMMS LAI data set^23^. The Global Inventory Modeling and Mapping Studies (GIMMS) LAI is derived from Advanced Very High Resolution Radiometer (AVHRR) satellite time series of the third generation of Normalized Difference Vegetation Index (NDVI3g). It is available at 15-day intervals and 8-km spatial resolution for July 1981 to December 2011. The GIMMS LAI is the only dataset providing time series long enough and with enough temporal resolution to allow the study of trends over the period considered in our study. Furthermore, interannually, GIMMS LAI data were significantly related (*P* < 0.01) to MODIS LAI (version C5) data for our 23 forests at a resolution of 1 km. The principles used for the generation of the GIMMS LAI data set were based on the use of neural networks that were first trained with data from the overlapping GIMMS NDVI3g and MODIS LAI products. The trained neural network algorithm was then applied using the land-cover class, the latitude and longitude coordinates and the NDVI3g as the input data to generate the full temporal coverage of the GIMMS LAI data set. Further details of the algorithm and quality assessment of GIMMS LAI data set given by Zhu *et al*.^23^.

#### Climate and weather data

We extracted the climatological mean annual temperature and precipitation (MATc, MAPc) for all forests from the WorldClim database, with a spatial resolution of around 1 km at the equator. Because time series of temperature and precipitation data from eddy covariance towers were of insufficient quality (too many missing values) for many of our forests, we opted to use the CRU TS3.21 data set^26^ from the Climatic Research Unit to extract temperature and precipitation time series for our forests as weather data. In addition, the SPEI (Standardized Precipitation-Evapotranspiration Index, Vicente-serrano *et al*.^27^ from the global SPEI database (http://sac.csic.es/spei/database.html) was used as a measure of drought intensity. Annual means of temperature (MAT), precipitation (MAP) and SPEI were calculated for each year. We also calculated annual values of MAT, MAP and SPEI for the warm half of the year (April – September) to be tested in the models as done for annual values.

#### Atmospheric CO_2_ concentrations

We used atmospheric CO_2_ concentrations recorded by eddy-covariance towers above the canopies of the forests when available. Annual atmospheric CO_2_ records, however, sometimes contain implausible values because of gaps along the time series (years with lower CO_2_ concentrations than the year before, higher than the next year’s or increases much larger than the normal increase of ~2 ppm per year recorded worldwide). We deleted the erroneous annual values and where possible filled the gaps using generalized additive models (GAM), adjusting a smoothing function. When this procedure was not possible, we used atmospheric CO_2_ concentration data from the Mauna Loa observatory, provided by the Scripps Institution of Oceanography (Scripps CO_2_ program). Original CO_2_ records from Mauna Loa and from individual forests were highly correlated (*P* < 0.0001) and their trend was very close to one (1.012 ± 0.005) using a zero intercept mixed model with random slopes. Therefore, using Mauna Loa’s data instead of original CO_2_ records from the eddy covariance towers could not influence the outcome of our results.

#### Deposition data

Annual data for N (NO3− + NH4+) and S (SO4−) wet deposition were extracted from the European Monitoring and Evaluation Programme (EMEP) with a spatial resolution of 0.15 × 0.15° for longitude and latitude, the MSC-W chemical transport model developed to estimate regional atmospheric dispersion and deposition of acidifying and eutrophying compounds of N and S over Europe and the National Atmospheric Deposition Program (NADP) covering the USA with a spatial resolution of 0.027 × 0.027° for longitude and latitude. We used only data for wet deposition because the NADP database did not contain records for dry deposition. Analyses were restricted to Europe and the USA because temporal gridded maps of atmospheric deposition were not available for other regions.

### Statistical analyses

#### Trends of individual forests

To test whether GPP, Re, NEP, LAI, N and S deposition, MAT and SPEI had changed during the study period, we first analysed the individual (for each forest) annual time series of each of these variables. The trends were extracted using the Theil-Sen slope estimator that minimizes the influence of extreme values (the breakdown point is ca. 29%) when calculating the trends (mblm package^58^ in R statistical software). This analysis has proven to be robust against temporal autocorrelation, non-normality and heteroscedasticity and produces results very similar to those of ordinary least squares regressions when errors are normally distributed and no outliers are present^59, 60^. Kernel densities were estimated to illustrate the proportions of forests with increasing and decreasing trends. Bootstrapping was used to statistically test whether the distribution of positive and negative trends across the forests was significantly different than the distribution of trends we would find by chance. We then tested the average trends (over all studied forests) in the studied variables using mixed models with random slopes (e.g., NEP ~ year) where the forest was the random factor (affecting the slopes of the year, therefore the trend). These mixed models also accounted for temporal autocorrelation using an autoregressive moving average (ARMA) (p = 1, q = 0) correlation structure. The average trends shown in the results section, and their significance, were calculated using the mixed effects models explained above.

To account for the spatial variability among forests (N = 23) in the trends of NEP, GPP, Re and LAI we used weighted linear models (adjusted by ordinary least squares and weighting for the number of observations for each forest) and stepwise forward model selection. The predictor variables we tested were climate (MATc and MAPc), mean S and N annual deposition rates, stand age, leaf type and habit, soil pH, the observed trends in LAI, S and N deposition, MAT, SPEI and the increase in CO_2_ since the beginning of the C-flux measurements. To further test that the observed trends in C fluxes were dependent on the age of the stand, we calculated a surrogate of the state of maturity of the forests by dividing the mean stand age by the logging maturity tree age as described by Stokland *et al*.^60^ for average productivity classes and included this variable as a predictor in the model. We also included the first-order interaction between pH and trends in N and S to test whether the effect of deposition depended on pH and vice-versa. We checked for multicolinearity overseeing the variance inflation factor. The variance explained by each variable within these models was assessed using the proportional marginal variance decomposition (PMVD) metric from the relaimpo R package^61^.

#### Temporal contributions and sensitivities of changes in C fluxes

The temporal contribution of each variable to the observed trends in GPP, Re, NEP and LAI was assessed using Generalised Linear Mixed Models (GLMMs) and model averaging (multi-model inference)^62^. This technique (GLMMs) allows disentangling the effect of one single predictor, while taking into account the variance shared (or correlation) with the other predictors. Model averaging is a statistical technique based on multi-model inference that calculates an average model with the estimates of the models that best fit the data while weighting their importance using the difference of the second-order Akaike Information Criterion (AICc) between each model and the model with lowest AICc. Using the forest as the random effect and an ARMA (p = 1, q = 0) autocorrelation structure, we fitted the saturated models as: response (annual anomalies) ~ (mean S deposition + S anomalies + CO_2_) + (mean N deposition + N anomalies + CO_2_) + (MATc + MAT anomalies + CO_2_) + (MAPc + SPEI + CO_2_) + mean S deposition x mean N deposition + MATc x climatic MAPc + CO_2_ x mean stand age, where variables between brackets where those for which we tested for first order interactions. Anomalies were calculated as the difference between the average value (e.g., MATc) and the annual value of a given year (i.e., MATan = MATc + MAT). When including the interactions between the climatic annual mean and the anomalies (MATc x MATan), we are including a changing effect of increasing or decreasing the anomalies depending on the mean for the forest (e.g., increasing temperature may have a positive effect in cold climates but a negative effect in warmer climates). In C flux models, we also included the anomalies of maximum LAI as a covariate. In our case, LAI can be interpreted as a surrogate for forest management, which implies that the reported effects of increasing CO_2_ concentrations are disconnected from any changes in forest structure (LAI or crown cover closure). Additionally, we fitted the saturated models using past N and S deposition, i.e. the cumulative totals of the previous 5 years, to test whether cumulative atmospheric deposition could improve prediction of interannual variability of C flux trends.

Using the model-averaging method [MuMIn R package] we fitted the saturated models for GPP, Re, NEP and LAI to construct an average model from the best models nested into the saturated models. 758246 models were calculated for each C flux and 379055 models for LAI. Average models were calculated using those models differing by less than four AICc units (in comparison with the best model) and fitted using restricted maximum likelihood. When calculating the model average estimates of the variables, estimates were replaced with 0 for models in which the explanatory variable was not included. Model residuals met the assumptions of normality, homocedasticity and linearity in all analyses.

We then used the average models to predict the change of the response variables during the study period (1995–2011). With the average models, we first calculated the observed trend (slope estimate ± standard error of the slope estimate) in our data using GLMMs with random slopes and temporal autocorrelation structure (ARMA, p = 1, q = 0). We then calculated the trend predicted by the average model and the trends predicted by the same model but maintaining the predictors constant one at a time (e.g., S deposition anomalies are held constant, using the median values per forest, while all other predictors change according to the observations). The difference between the predictions for the whole model and when one variable was controlled was the contribution of that predictor variable to the change in the response variable. The difference between all individual contributions and the observed trend were considered to be unknown contributions. Finally, we calculated the average NEP, GPP and Re sensitivities to predictor changes dividing the temporal contributions by the trends of the predictor variables. All errors were calculated using the error-propagation method.

## Electronic supplementary material

Supplementary Information 
