# Supplementary material for: Atmospheric deposition, CO2, and change in the land carbon sink
Source: Sci Rep. 2017 Aug 29;7:9632. doi: 10.1038/s41598-017-08755-8 (PMC5574890; doi:10.1038/s41598-017-08755-8)
Supplement: Supplementary file 1 — Supplementary Information [file 41598_2017_8755_MOESM1_ESM.pdf]

Supplementary Information

Atmospheric deposition, CO_2, and change in the land carbon sink

Authors: M. Fernández-Martínez[*1,2], S. Vicca[3], I. A. Janssens[3], P. Ciais[4], M. Obersteiner[5], M. Bartrons[1,2], J. Sardans[1,2], A. Verger[1,2], J. G. Canadell[6], F. Chevallier[4], X. Wang[7,8], C. Bernhofer[9], P.S. Curtis[10], D, Gianelle[11,12], T. Grünwald[9], B. Heinesch[13], A. Ibrom[14], A. Knohl[15], T. Laurila[16], B.E. Law[17], J.M. Limousin[18], B. Longdoz[19], D. Loustau[20], I. Mammarella[21], G. Matteucci[22,23], R.K. Monson[24], L. Montagnani[25,26], E.J. Moors[27,28], J.W. Munger[29], D. Papale[30], S.L. Piao[7,31], J. Peñuelas[1,2].

Figure captions

Fig. S1. Map indicating the locations of the 23 forest sites with eddy-covariance data. The forests were located in temperate and boreal biomes across Europe and the USA. The boxes approximately indicate the extent of the analysis (Europe and the USA). The map was created using R software (version 3.1.1, URL http://www.R-project.org) and "mapdata" package (mapdata: Extra Map Databases, version 2.2), using free vector map data from Natural Earth (URL http://www.naturalearthdata.com).

Fig. S2. Plots showing the relationships between the individual trends in C fluxes and the trend of S deposition in the 23 studied forests.

Table S1. Rates of change in predictors per site. Individual Trends were computed using the robust Theil-Sen slope estimator. *P* indicates a two-tailed *P* (H1: trend ≠0). Units are ppm for CO_2, kg ha^{-1} $yr^{-1}$for average N (nitrogen) and S (sulphur) deposition, kg ha^{-1} yr^{-2} for N and S deposition trends and, K yr^{-1} for temperature and standard deviation yr^{-1} for SPEI. Abbreviations: TS, Theil-Sen; for CO_2 source, ML indicates Mauna Loa and EC eddy covariance tower.

Table S2. Average rates of change in predictors for 1995–2011. Trends were calculated using GLMMs with random slopes, with the forest as a random effect and year as a fixed effect. Models also used an ARMA (1,0) autocorrelation structure. See Methods further details. Trend units are ppm yr^{-1} for CO_2, kg ha^{-1} yr^{-2} for N and S deposition, K yr^{-1} for temperature and standard deviation yr^{-1} for SPEI.

Fig. S1

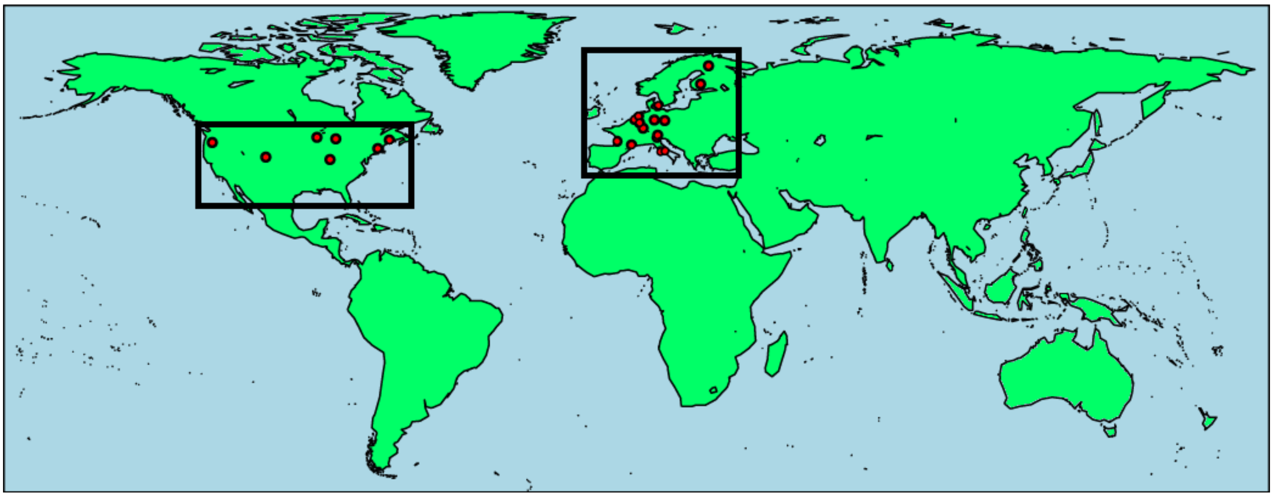

Fig S2

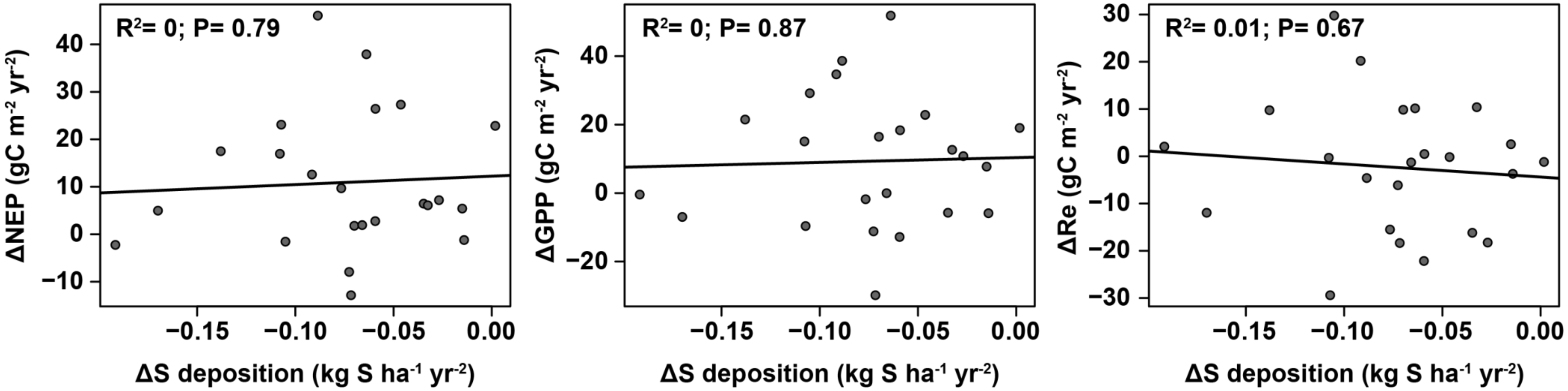

R²= 0; P= 0.79
ΔNEP (gC m⁻² yr⁻²)
ΔS deposition (kg S ha⁻¹ yr⁻²)
R²= 0; P= 0.87
ΔGPP (gC m⁻² yr⁻²)
ΔS deposition (kg S ha⁻¹ yr⁻²)
R²= 0.01; P= 0.67
ΔRe (gC m⁻² yr⁻²)
ΔS deposition (kg S ha⁻¹ yr⁻²)

Table S1

**Forest**	**Source CO_2**	**ΔCO_2**	**N**	**S**	**N TS Trend**	***P***	**S TS Trend**	***P***	**MAT TS Trend**	***P***
Brasschaat	ML	27.9	11.64 ± 0.39	2.29 ± 0.18	-0.23 ± 0.07	0.0215	-0.14 ± 0.02	0.0002	0.02 ± 0.02	0.3513
Castelporziano	EC	18.9	6.33 ± 0.57	1.28 ± 0.15	0.06 ± 0.19	0.8580	-0.06 ± 0.05	0.5915	0.00 ± 0.02	1.0000
Collelongo	ML	30.1	5.66 ± 0.20	1.14 ± 0.12	-0.04 ± 0.04	0.3502	-0.07 ± 0.01	0.0018	0.01 ± 0.02	0.2758
Hainich	EC	31.7	8.72 ± 0.38	1.15 ± 0.09	-0.24 ± 0.10	0.1148	-0.07 ± 0.01	0.0032	-0.01 ± 0.04	0.9453
Harvard	EC	36.3	6.23 ± 1.17	4.18 ± 0.32	-0.21 ± 0.04	0.0000	-0.09 ± 0.04	0.0644	0.06 ± 0.03	0.0104
Hesse	EC	35.2	8.76 ± 0.24	1.38 ± 0.08	0.07 ± 0.06	0.4285	-0.06 ± 0.01	0.0008	0.03 ± 0.02	0.4285
Howland MT	EC	22.6	3.00 ± 0.11	3.16 ± 0.12	-0.04 ± 0.02	0.1606	-0.03 ± 0.03	0.5022	-0.02 ± 0.06	0.8548
Howland F	EC	14.4	20.83 ± 0.13	2.91 ± 0.16	-0.04 ± 0.05	0.5334	-0.02 ± 0.06	1.0000	-0.03 ± 0.08	0.6404
Hyytiala	EC	37.4	2.83 ± 0.08	0.66 ± 0.05	-0.01 ± 0.02	0.8431	-0.03 ± 0.01	0.0003	0.05 ± 0.04	0.2350
Lavarone	EC	47.2	16.89 ± 0.32	1.14 ± 0.09	-0.14 ± 0.13	0.1753	-0.09 ± 0.01	0.0012	-0.05 ± 0.07	0.4655
Le Bray	EC	13.2	4.90 ± 0.37	1.16 ± 0.08	0.17 ± 0.10	0.1611	-0.03 ± 0.03	0.5334	-0.04 ± 0.04	0.1844
Loobos	EC	32.7	13.26 ± 0.51	1.80 ± 0.13	-0.33 ± 0.07	0.0041	-0.11 ± 0.02	0.0001	0.03 ± 0.02	0.4884
Metolius	EC	28.1	0.48 ± 0.02	0.38 ± 0.02	-0.01 ± 0.01	0.5915	0.00 ± 0.01	0.8580	-0.15 ± 0.04	0.0200
Morgan Monroe	EC	25.1	6.57 ± 0.29	6.03 ± 0.33	-0.05 ± 0.09	0.5830	-0.19 ± 0.06	0.0124	0.04 ± 0.04	0.2215
Niwot ridge	ML	21.5	3.46 ± 0.28	1.25 ± 0.10	-0.16 ± 0.07	0.0467	-0.07 ± 0.02	0.0075	-0.06 ± 0.03	0.0467
Park Falls	EC	31.2	4.48 ± 0.32	2.17 ± 0.16	-0.09 ± 0.05	0.1889	-0.08 ± 0.02	0.0160	-0.01 ± 0.05	0.9128
Puechabon	ML	25.4	6.18 ± 0.30	1.20 ± 0.10	-0.17 ± 0.07	0.2758	-0.07 ± 0.02	0.0617	0.02 ± 0.05	0.9378
Renon	EC	33.8	10.18 ± 0.25	0.83 ± 0.09	-0.10 ± 0.06	0.0769	-0.06 ± 0.01	0.0000	-0.03 ± 0.04	0.4277
Sodankyla	EC	28.8	1.12 ± 0.05	0.26 ± 0.02	0.00 ± 0.01	0.9453	-0.01 ± 0.00	0.0020	0.04 ± 0.07	0.6800
Soroe	EC	38.7	7.72 ± 0.24	1.25 ± 0.07	-0.02 ± 0.05	0.6693	-0.05 ± 0.01	0.0019	0.05 ± 0.03	0.0995
Tharandt	EC	27.2	9.72 ± 0.32	1.65 ± 0.15	-0.18 ± 0.06	0.0294	-0.11 ± 0.02	0.0001	0.04 ± 0.03	0.2763
UMBS	EC	26.7	4.79 ± 0.19	3.05 ± 0.20	-0.12 ± 0.04	0.0240	-0.17 ± 0.02	0.0012	0.02 ± 0.05	0.9514
Vielsalm	ML	23	10.07 ± 0.38	1.75 ± 0.13	-0.27 ± 0.07	0.0124	-0.11 ± 0.01	0.0001	0.03 ± 0.03	0.0995

Table S2

	Mean	SE	Total change 1995–2011	SE	*P*
**CO_2**	2.043	0.139	34.731	2.370	<0.001
**Nitrogen**	-0.087	0.017	-1.478	0.294	<0.001
**Sulphur**	-0.086	0.008	-1.464	0.138	<0.001
**Temperature**	0.003	0.008	0.047	0.136	0.73
**SPEI**	-0.001	0.005	-0.011	0.091	0.91

Summary of models and statistical analyses

1. Spatial variability of trends in C fluxes: relationships between annual trends in carbon fluxes and predictors

Models were adjusted using stepwise forward models using the following saturated model: C flux trend ~ MATc + MAPc + N dep + S dep + Age + corrected maturity age + leaf type + (ph + n.wet.t + s.wet.t)^2 + n.wet.t + deltacdioxide + MAT.t + SPEI.t + lai.max.t + Total Biomass, where ^2 indicates a first-order interaction of the variables within the brackets. Models were weighted for the n years of the plots. ".t" suffixes in variables indicate the trends of the variable. Significance levels: $^{(*)}$, $P < 0.1$; *, $P < 0.05$; **, $P < 0.01$; ***, $P < 0.001$.

GPP

	Estimate	SE	Beta	SE	*t*	Pr(>|t|)		PMVD
**(Intercept)**	-77.6300	15.5900	0.0000	0.0000	-4.979	0.000252	***	
**deltacdioxide**	2.1190	0.4382	0.7630	0.1578	4.836	0.000325	***	0.40
**Total Biomass**	0.0027	0.0006	0.6834	0.1600	4.271	0.000912	***	0.31
**R^2**	0.71	**R^2 adj**	0.66					

Re

	Estimate	SE	Beta	SE	*t*	Pr(>|t|)		PMVD
**(Intercept)**	-11.1942	11.1889	0.0000	0.0000	-1.000	0.336826		
**Total Biomass**	0.0041	0.0009	1.1697	0.2511	4.658	0.000553	***	0.37
**MAT.t**	228.3477	63.5489	0.7543	0.2099	3.593	0.003691	**	0.20
**Corrected Mat. Age**	-38.7487	15.8033	-0.5851	0.2386	-2.452	0.030485	*	0.11
**R^2**	0.68	**R^2 adj**	0.60					

NEP

	Estimate	SE	Beta	SE	*t*	Pr(>|t|)		PMVD
**(Intercept)**	-36.1746	7.3628	0.0000	0.0000	-4.913	9.65E-05	***	
**deltacdioxide**	1.4555	0.2473	0.7462	0.1268	5.885	1.15E-05	***	0.43
**N dep**	1.1745	0.3706	0.3943	0.1244	3.169	0.00505	**	0.14
**MAT.t**	-135.7225	39.6104	-0.4430	0.1293	-3.426	0.00283	**	0.14
**R^2**	0.71	**R^2 adj**	0.66					

LAI max

	Estimate	SE	Beta	SE	*t*	Pr(>|t|)	PMVD
**(Intercept)**	-0.1257	0.0427	0.0000	0.0000	-2.941	0.00807	
**pH**	0.0293	0.0084	0.5295	0.1520	3.483	0.00235	0.38
**MAT.t**	-0.3992	0.1849	-0.3345	0.1549	-2.159	0.04315	0.15
**R^2**	0.53	**R^2 adj**	0.48				

2. Models to assess temporal contributions: relationships between C fluxes and predictor annual values using model averaging of generalized mixed models (only models with ΔAICc <4).

2.1 – Model averaging using inteannual data from the 23 forests

Saturated models' formulation: Response variable ~ maximum lai anomalies + (mean S deposition + S anomalies + CO_2)^2 + (mean N deposition + N anomalies + CO_2)^2 + (MATc + MAT anomalies + CO_2)^2 + (MAPc + SPEI + CO_2)^2 + mean S deposition * mean N deposition + MATc * MAPc + age * CO_2, where ^2 indicates a first-order interaction of the variables within the brackets. SE, standard error; Rel. Imp, relative importance of the predictors. Significance levels: $^{(*)}$, $P < 0.1$; *, $P < 0.05$; **, $P < 0.01$; ***, $P < 0.001$.

List of acronyms

age: Stand age

cdioxide.an: CO_2 concentration anomalies

lai.max.an: Maximum LAI anomalies

ndep: Mean N deposition

sdep: Mean S deposition

n.wet.an: N deposition anomalies

s.wet.an: S deposition anomalies

mat: climatic mean annual temperature

map: climatic mean annual precipitation

tmean.an: mean annual temperature anomalies

spei: Standardized Precipitation Evapotranspiration Index

2.1.1 Average model for NEP (for years 1995 - 2011)

Variable	Estimate	SE	Adj. SE	Rel. Imp	z value	Pr(>|z|)	
(Intercept)	11.420	24.240	24.350		0.47	0.6391	
age	-0.074	0.179	0.191	1.00	0.39	0.6973	
cdioxide.an	6.825	2.290	2.300	1.00	2.97	0.0030	**
lai.max.an	-1.413	6.981	7.006	0.14	0.20	0.8401	
n.wet.an	-7.667	14.370	14.390	0.39	0.53	0.5943	
ndep	0.018	1.876	1.999	0.97	0.01	0.9929	
s.wet.an	20.320	19.860	19.920	0.99	1.02	0.3078	
sdep	0.718	3.030	3.191	0.20	0.23	0.8219	
map	0.002	0.017	0.018	0.11	0.11	0.9157	
spei	-1.396	7.512	7.540	0.15	0.19	0.8531	
mat	0.067	0.769	0.818	0.11	0.08	0.9350	
tmean.an	-13.340	11.190	11.220	0.77	1.19	0.2344	
age:cdioxide.an	-0.064	0.019	0.019	1.00	3.29	0.0010	***
cdioxide.an:map	0.000	0.001	0.001	0.01	0.05	0.9620	
cdioxide.an:mat	-0.002	0.034	0.034	0.01	0.06	0.9561	
cdioxide.an:n.wet.an	-0.015	0.171	0.171	0.03	0.09	0.9291	
cdioxide.an:ndep	0.536	0.222	0.223	0.97	2.40	0.0162	*
cdioxide.an:s.wet.an	3.040	1.285	1.291	0.99	2.36	0.0185	*
cdioxide.an:sdep	-0.007	0.098	0.098	0.01	0.07	0.9454	
cdioxide.an:spei	0.009	0.202	0.203	0.01	0.05	0.9641	
cdioxide.an:tmean.an	-0.025	0.302	0.304	0.08	0.08	0.9335	
ndep:n.wet.an	0.533	1.273	1.275	0.21	0.42	0.6760	
ndep:sdep	0.002	0.160	0.172	0.01	0.01	0.9888	
sdep:s.wet.an	0.253	1.947	1.951	0.03	0.13	0.8970	

57 with models ΔAICc < 4

2.1.2 Average model for GPP (for years 1995 - 2011)

Variable	Estimate	SE	Adj. SE	Rel.Imp	z value	Pr(>|z|)	
(Intercept)	-9.272	27.020	27.140		0.342	0.7326	
age	-0.001	0.074	0.079	0.09	0.02	0.9869	
cdioxide.an	5.120	2.138	2.144	1.00	2.39	0.0169	*
lai.max.an	0.619	7.112	7.142	0.08	0.09	0.9309	
n.wet.an	9.598	11.720	11.760	0.71	0.82	0.4142	
ndep	-0.072	0.869	0.919	0.12	0.08	0.9374	
s.wet.an	-34.990	27.690	27.780	0.92	1.26	0.2079	
sdep	-0.007	2.101	2.224	0.08	0.00	0.9973	
map	0.001	0.021	0.022	0.10	0.03	0.9740	
spei	16.000	31.420	31.480	0.42	0.51	0.6113	
mat	0.574	1.901	1.989	0.28	0.29	0.7728	
tmean.an	15.790	18.100	18.140	0.73	0.87	0.3841	
age:cdioxide.an	0.000	0.004	0.004	0.01	0.08	0.9350	
cdioxide.an:map	0.000	0.001	0.001	0.01	0.04	0.9663	
cdioxide.an:mat	-0.063	0.223	0.224	0.11	0.28	0.7791	
cdioxide.an:n.wet.an	-0.677	1.092	1.093	0.37	0.62	0.5360	
cdioxide.an:ndep	0.014	0.089	0.089	0.04	0.15	0.8792	
cdioxide.an:s.wet.an	-2.775	2.448	2.453	0.70	1.13	0.2578	
cdioxide.an:sdep	0.002	0.060	0.061	0.00	0.03	0.9737	
cdioxide.an:spei	-0.037	0.516	0.518	0.03	0.07	0.9430	
cdioxide.an:tmean.an	0.339	0.941	0.943	0.21	0.36	0.7193	
map:spei	-0.002	0.022	0.022	0.01	0.08	0.9338	
mat:tmean.an	0.274	1.527	1.530	0.05	0.18	0.8579	

233 models with ΔAICc < 4

2.1.3 Average model for Re (for years 1995 - 2011)

Variable	Estimate	SE	Adj. SE	Rel.Imp	z value	Pr(>|z|)	
**(Intercept)**	-18.530	27.210	27.330		0.678	0.4977	
**age**	0.003	0.071	0.075	0.088	0.04	0.9721	
**cdioxide.an**	0.507	2.912	2.920	1.000	0.17	0.8622	
**lai.max.an**	8.362	17.610	17.650	0.314	0.47	0.6356	
**ndep**	-0.023	0.642	0.682	0.071	0.03	0.9729	
**n.wet.an**	19.640	12.050	12.090	0.896	1.62	0.1043	
**sdep**	-0.363	3.764	3.987	0.268	0.09	0.9275	
**s.wet.an**	-65.780	26.510	26.620	1.000	2.47	0.0135	*
**mat**	0.695	2.328	2.461	0.550	0.28	0.7778	
**tmean.an**	32.250	22.350	22.410	1.000	1.44	0.1502	
**map**	-0.001	0.019	0.020	0.081	0.04	0.9709	
**spei**	26.350	28.090	28.150	0.642	0.94	0.3492	
**age:cdioxide.an**	0.000	0.004	0.004	0.014	0.08	0.9332	
**cdioxide.an:n.wet.an**	-0.014	0.290	0.291	0.068	0.05	0.9628	
**cdioxide.an:sdep**	0.309	0.772	0.773	0.181	0.40	0.6889	
**cdioxide.an:s.wet.an**	-6.568	1.760	1.768	1.000	3.72	0.0002	***
**cdioxide.an:mat**	-0.321	0.463	0.464	0.422	0.69	0.4883	
**cdioxide.an:tmean.an**	1.172	1.466	1.469	0.547	0.80	0.4251	
**cdioxide.an:map**	0.000	0.001	0.001	0.003	0.04	0.9718	
**cdioxide.an:spei**	-0.068	0.665	0.668	0.061	0.10	0.9184	
**sdep:s.wet.an**	0.025	1.195	1.200	0.010	0.02	0.9835	
**mat:tmean.an**	1.214	2.966	2.971	0.219	0.41	0.6828	

181 models with ΔAICc < 4

2.1.4 Average model for LAI (for years 1995 - 2011)

**Variable**	**Estimate**	**SE**	**Adj. SE**	**Rel.Imp**	**z value**	**Pr(>|z|)**	
**(Intercept)**	0.030	0.096	0.096		0.31	0.754	
**age**	0.000	0.000	0.000	0.36	0.18	0.854	
**cdioxide.an**	0.018	0.015	0.015	1.00	1.22	0.223	
**map**	0.000	0.000	0.000	0.23	0.19	0.848	
**spei**	0.002	0.039	0.039	0.09	0.06	0.952	
**mat**	0.000	0.007	0.007	0.74	0.01	0.992	
**tmean.an**	-0.038	0.045	0.045	0.77	0.84	0.402	
**ndep**	0.000	0.002	0.002	0.09	0.02	0.981	
**n.wet.an**	0.001	0.009	0.009	0.11	0.07	0.945	
**sdep**	-0.012	0.018	0.019	0.89	0.63	0.528	
**s.wet.an**	-0.098	0.093	0.094	0.98	1.04	0.298	
**age:cdioxide.an**	0.000	0.000	0.000	0.31	0.58	0.565	
**cdioxide.an:map**	0.000	0.000	0.000	0.14	0.36	0.721	
**cdioxide.an:mat**	0.002	0.001	0.001	0.73	1.26	0.209	
**cdioxide.an:tmean.an**	-0.004	0.004	0.004	0.59	0.92	0.360	
**cdioxide.an:sdep**	-0.007	0.004	0.004	0.89	1.89	0.059	.
**cdioxide.an:s.wet.an**	-0.011	0.005	0.005	0.96	2.10	0.036	*
**s.wet.an:sdep**	0.004	0.015	0.015	0.18	0.27	0.787	
**map:spei**	0.000	0.000	0.000	0.01	0.06	0.953	
**mat:tmean.an**	0.001	0.004	0.004	0.10	0.19	0.852	

103 models with ΔAICc < 4

2.2. Results from the saturated (i.e., full) models (model summaries and temporal contributions) using temperature and SPEI for the warm half of the year (April – September) for the 23 forests.

NEP

	Value	Std.Error	DF	t-value	p-value
**(Intercept)**	25.26972	92.60895	250	0.272865	0.7852
**lai.max.an**	-13.9428	17.10382	250	-0.81519	0.4157
**age**	-0.08846	0.20282	14	-0.43614	0.6694
**cdioxide.an**	7.843715	5.1522	250	1.5224	0.1292
**sdep**	-3.33984	33.42586	14	-0.09992	0.9218
**s.wet.an**	10.18673	53.0002	250	0.192202	0.8477
**ndep**	-0.14425	6.85113	14	-0.02106	0.9835
**n.wet.an**	-26.7314	18.71101	250	-1.42864	0.1544
**mat**	1.342473	14.50814	14	0.092532	0.9276
**temp.warm.an**	-9.8591	19.28865	250	-0.51113	0.6097
**map**	-0.02279	0.1408	14	-0.16182	0.8738
**spei.hot**	21.19634	66.55602	250	0.318474	0.7504
**age:cdioxide.an**	-0.05766	0.02085	250	-2.76498	0.0061
**sdep:s.wet.an**	2.568747	9.23095	250	0.278276	0.781
**cdioxide.an:sdep**	-0.62546	0.949	250	-0.65907	0.5105
**cdioxide.an:s.wet.an**	3.690413	1.82765	250	2.019212	0.0445
**ndep:n.wet.an**	2.103415	1.7637	250	1.192614	0.2342
**cdioxide.an:ndep**	0.653451	0.23053	250	2.83462	0.005
**cdioxide.an:n.wet.an**	-0.81433	0.92146	250	-0.88373	0.3777
**mat:temp.warm.an**	-1.72551	2.32907	250	-0.74086	0.4595
**cdioxide.an:mat**	0.049929	0.3242	250	0.154004	0.8777
**cdioxide.an:temp.warm.an**	-0.48956	1.12629	250	-0.43466	0.6642
**map:spei.hot**	-0.03304	0.07926	250	-0.41688	0.6771
**cdioxide.an:map**	-0.00116	0.00674	250	-0.172	0.8636
**cdioxide.an:spei.hot**	1.309095	1.72203	250	0.760206	0.4478
**sdep:ndep**	0.05407	1.80042	14	0.030032	0.9765
**sdep:map**	0.005158	0.03464	14	0.148915	0.8837
**mat:map**	-0.0008	0.01802	14	-0.04414	0.9654
**Phi**	**Rsqm**	**Rsqc**	**AICc**	**BIC**	**logLik**
0.2954446	0.242374	0.242374	3546.102	3652.451	-1738.24

2.2.1 – NEP Temporal contributions

	**mean y^{-1}**	**SE**	***t***	***P***
**Data trend**	**7.599**	**2.099**	**3.62000**	**0.00040**
**cdioxide**	**10.640**	**1.090**	**9.75797**	**0.00000**
**n.wet.an**	0.775	1.520	0.50941	0.30710
**s.wet.an**	**-1.985**	**1.411**	**-1.40674**	**0.08489**
**tmean.an**	-1.015	1.502	-0.67621	0.25204
**spei**	0.130	1.500	0.08672	0.46574
**LAI**	-0.357	1.489	-0.23980	0.40606
**Unknown**	-0.588	4.077	-0.14418	0.44316

2.2.2 – GPP

	Value	Std.Error	DF	t-value	p-value
**(Intercept)**	15.1771	124.9807	250	0.121436	0.9034
**lai.max.an**	0.05699	24.90648	250	0.002288	0.9982
**age**	-0.01349	0.27361	14	-0.0493	0.9614
**cdioxide.an**	11.77647	7.11432	250	1.655319	0.0991
**sdep**	-7.17418	45.15145	14	-0.15889	0.876
**s.wet.an**	-74.7771	76.54979	250	-0.97684	0.3296
**ndep**	-1.43882	9.26116	14	-0.15536	0.8788
**n.wet.an**	7.18204	27.33785	250	0.262714	0.793
**mat**	2.86488	19.61705	14	0.146041	0.886
**temp.warm.an**	57.18129	28.53551	250	2.003864	0.0462
**map**	-0.03458	0.19014	14	-0.18187	0.8583
**spei.hot**	206.3117	97.88954	250	2.107597	0.0361
**age:cdioxide.an**	-0.05562	0.02882	250	-1.93013	0.0547
**sdep:s.wet.an**	6.93231	13.3947	250	0.517542	0.6052
**cdioxide.an:sdep**	2.1749	1.32897	250	1.636533	0.103
**cdioxide.an:s.wet.an**	-3.17238	2.63689	250	-1.20308	0.2301
**ndep:n.wet.an**	0.90267	2.5985	250	0.347381	0.7286
**cdioxide.an:ndep**	0.51316	0.32177	250	1.594812	0.112
**cdioxide.an:n.wet.an**	-0.94639	1.34532	250	-0.70347	0.4824
**mat:temp.warm.an**	-6.08596	3.46873	250	-1.75452	0.0806
**cdioxide.an:mat**	-0.87045	0.44943	250	-1.9368	0.0539
**cdioxide.an:temp.warm.an**	0.2967	1.66495	250	0.178203	0.8587
**map:spei.hot**	-0.19894	0.11673	250	-1.7042	0.0896
**cdioxide.an:map**	-0.00978	0.00927	250	-1.05477	0.2925
**cdioxide.an:spei.hot**	-0.16655	2.53177	250	-0.06578	0.9476
**sdep:ndep**	0.14508	2.43395	14	0.059605	0.9533
**sdep:map**	0.00852	0.04681	14	0.181913	0.8583
**mat:map**	-0.00079	0.02437	14	-0.03223	0.9747

Phi	Rsqm	Rsqc	AICc	BIC	logLik
0.2308718	0.220671	0.220671	3764.943	3871.291	-1847.66

Temporal contributions

	mean y-1	SE	*t*	*P*
**Data trend**	**10.386**	**2.854**	**3.63935**	**0.00030**
**cdioxide**	**9.308**	**1.330**	**6.99874**	**0.00000**
**n.wet.an**	-1.001	1.831	-0.54696	0.29423
**s.wet.an**	**4.498**	**1.594**	**2.82223**	**0.00419**
**tmean.an**	0.675	1.741	0.38795	0.35040
**spei**	1.790	2.453	0.72962	0.23564
**LAI**	0.001	1.766	0.00076	0.49970
**Unknown**	-4.886	5.289	-0.92377	0.18149

2.2.3 – Re

	Value	Std.Error	DF	t-value	p-value
**(Intercept)**	-12.1282	121.3939	250	-0.09991	0.9205
**lai.max.an**	15.39452	23.30676	250	0.660517	0.5095
**age**	0.07416	0.26581	14	0.279013	0.7843
**cdioxide.an**	4.39585	6.83368	250	0.643262	0.5206
**sdep**	-2.5121	43.83465	14	-0.05731	0.9551
**s.wet.an**	-85.2725	71.92902	250	-1.18551	0.2369
**ndep**	-1.70817	8.98793	14	-0.19005	0.852
**n.wet.an**	34.17782	25.53889	250	1.338266	0.182
**mat**	0.87198	19.0355	14	0.045808	0.9641
**temp.warm.an**	66.02805	26.4901	250	2.492555	0.0133
**map**	-0.00787	0.18462	14	-0.04262	0.9666
**spei.hot**	181.3342	91.1403	250	1.989616	0.0477
**age:cdioxide.an**	-0.0013	0.02767	250	-0.04704	0.9625
**sdep:s.wet.an**	4.50885	12.55709	250	0.359068	0.7198
**cdioxide.an:sdep**	2.87246	1.26747	250	2.266287	0.0243
**cdioxide.an:s.wet.an**	-7.00665	2.4785	250	-2.82697	0.0051
**ndep:n.wet.an**	-1.30446	2.41737	250	-0.53962	0.5899
**cdioxide.an:ndep**	-0.16081	0.30741	250	-0.52312	0.6014
**cdioxide.an:n.wet.an**	0.07522	1.25717	250	0.059829	0.9523
**mat:temp.warm.an**	-4.09729	3.20912	250	-1.27676	0.2029
**cdioxide.an:mat**	-0.90676	0.43085	250	-2.10459	0.0363
**cdioxide.an:temp.warm.an**	1.00636	1.54592	250	0.650974	0.5157
**map:spei.hot**	-0.1599	0.10861	250	-1.4722	0.1422
**cdioxide.an:map**	-0.00913	0.00892	250	-1.02256	0.3075
**cdioxide.an:spei.hot**	-1.67028	2.35747	250	-0.70851	0.4793
**sdep:ndep**	0.20576	2.36206	14	0.087112	0.9318
**sdep:map**	0.00105	0.04544	14	0.023075	0.9819
**mat:map**	0.00088	0.02365	14	0.037319	0.9708

Phi	Rsqm	Rsqc	AICc	BIC	logLik
0.263431	0.202652	0.202652	3726.29	3832.639	-1828.33

Temporal contributions

	mean y-1	SE	*t*	*P*
**Data trend**	2.792	2.818	0.99108	0.32250
**cdioxide**	-1.733	1.542	-1.12366	0.135034
**n.wet.an**	-1.561	2.087	-0.74765	0.230246
**s.wet.an**	**6.464**	**1.634**	**3.95578**	**0.000216**
**tmean.an**	1.585	1.937	0.81845	0.209777
**spei**	0.441	1.990	0.22161	0.41306
**LAI**	0.333	1.980	0.16795	0.433875
**Unknown**	-2.737	5.383	-0.50838	0.30745

Additional references (for Table 1)

1. de Pury DGG, Ceulemans R (1997) Scaling-up carbon fluxes from leaves to stands in a patchy coniferous/deciduous forest. In: Mohren GMJ (ed) Impacts of global change on tree and forest ecosystems. Kluwer Academic, Dordrecht, pp 263–272

2. Reichstein, M. *et al.* Ecosystem respiration in two Mediterranean evergreen Holm Oak forests: drought effects and decomposition dynamics. *Funct. Ecol.* **16**, 27–39 (2002).

3. Valentini, R. *et al.* Seasonal net carbon dioxide exchange of a beech forest with the atmosphere. *Glob. Chang. Biol.* **2,** 199–207 (1996).

4. Knohl, A., Schulze, E.-D., Kolle, O. & Buchmann, N. Large carbon uptake by an unmanaged 250-year-old deciduous forest in Central Germany. *Agric. For. Meteorol.* **118**, 151–167 (2003).

5. Urbanski, S. et al. Factors controlling CO_2 exchange on timescales from hourly to decadal at Harvard Forest. *J. Geophys. Res*. **112**, G02020 (2007).

6. Genet, H., Bréda, N. & Dufrêne, E. Age-related variation in carbon allocation at tree and stand scales in beech (Fagus sylvatica L.) and sessile oak (Quercus petraea (Matt.) Liebl.) using a chronosequence approach. *Tree Physiol*. **30**, 177–92 (2010).

7. Zhang, J., Wu, L., Huang, G. & Notaro, M. Relationships between large-scale circulation patterns and carbon dioxide exchange by a deciduous forest. *J. Geophys. Res*. **116**, D04102 (2011).

8. Kowalski, A. S. *et al*. Paired comparisons of carbon exchange between undisturbed and regenerating stands in four managed forests in Europe. *Glob. Chang. Biol*. **10**, 1707–1723 (2004).

9. Chiti, T. *et al*. Predicting changes in soil organic carbon in mediterranean and alpine forests during the Kyoto Protocol commitment periods using the CENTURY model. *Soil Use Manag.* **26**, 475–484 (2010).

10. Granier, A. et al. Evidence for soil water control on carbon and water dynamics in European forests during the extremely dry year: 2003. *Agric. For. Meteorol.* **143**, 123–145 (2007).

11. Richardson, A. D. *et al.* Influence of spring and autumn phenological transitions on forest ecosystem productivity. *Philos. Trans. R. Soc. Lond. B. Biol. Sci.* **365,** 3227–46 (2010).

12. Law, B. E., Baldocchi, D. D. & Anthoni, P. M. Below-canopy and soil CO2 fluxes in a ponderosa pine forest. *Agric. For. Meteorol.* **94,** 171–188 (1999).

13. Monson, R. K. *et al.* Carbon sequestration in a high-elevation, subalpine forest. *Glob. Chang. Biol.* **8,** 459–478 (2002)

14. Bakwin, P. S., Tans, P. P., Hurst, D. F. & Zhao, C. Measurements of carbon dioxide on very tall towers: results of the NOAA/CMDL program. *Tellus B* **50**, 401–415 (1998).

15. Montagnani, L. *et al.* A new mass conservation approach to the study of CO 2 advection in an alpine forest. *J. Geophys. Res*. **114**, D07306 (2009).

16. Louis, J. *et al.* Remote sensing of sunlight-induced chlorophyll fluorescence and reflectance of Scots pine in the boreal forest during spring recovery. *Remote Sens. Environ.* **96,** 37–48 (2005).

17. Pilegaard, K., Ibrom, A., Courtney, M.S., Hummelshøj, P. and Jensen, N.O., 2011. Increasing net CO2 uptake by a Danish beech forest during the period from 1996 to 2009. Agricultural and Forest Meteorology, 151: 934–946.

18. Grünwald, T. & Bernhofer, C. A decade of carbon, water and energy flux measurements of an old spruce forest at the Anchor Station Tharandt. *Tellus B* **59,** 387–396 (2007).

19. Rothstein, D. E., Zak, D. R., Pregitzer, K. S. & Curtis, P. S. Kinetics of nitrogen uptake by Populus tremuloides in relation to atmospheric CO2 and soil nitrogen availability. *Tree Physiol.* **20,** 265–270 (2000).

20. Longdoz, B., Yernaux, M. & Aubinet, M. Soil CO_2 efflux measurements in a mixed forest: impact of chamber disturbances, spatial variability and seasonal evolution. *Glob. Chang. Biol.* **6,** 907–917 (2000).
